# Proportion and number of incident cancer deaths in coronary artery disease

**DOI:** 10.1002/cam4.6595

**Published:** 2023-09-27

**Authors:** Jin Liu, Shiqun Chen, Yang Zhou, Haozhang Huang, Qiang Li, Yan Liang, Shaohong Dong, Xiaoyu Huang, Liling Chen, Xueyan Zheng, Ruilin Meng, Congzhuo Jia, Jiyan Chen, Ning Tan, Yong Liu

**Affiliations:** ^1^ Department of Cardiology Guangdong Cardiovascular Institute, Guangdong Provincial People's Hospital, Southern Medical University Guangzhou China; ^2^ Department of Guangdong Provincial Key Laboratory of Coronary Heart Disease Prevention Guangdong Provincial People's Hospital, Guangdong Academy of Medical Sciences Guangzhou China; ^3^ Global Health Research Center, Guangdong Provincial People's Hospital Guangdong Academy of Medical Science Guangzhou China; ^4^ The Second School of Clinical Medicine Southern Medical University Guangzhou China; ^5^ Department of Cardiology Maoming People's Hospital Maoming China; ^6^ Department of Cardiology Shenzhen People's Hospital Shenzhen China; ^7^ Department of Cardiology Yangjiang People's Hospital Yangjiang Guangdong P.R. China; ^8^ Department of Cardiology Longyan First Affiliated Hospital of Fujian Medical University Longyan Fujian P.R. China; ^9^ Institute of Control and Prevention for Chronic Non‐Infective Disease, Guangdong Provincial Center for Disease Control and Prevention Guangzhou China; ^10^ Guangdong Provincial People's Hospital, School of Medicine South China University of Technology Guangzhou China

**Keywords:** cancer‐specific mortality, cardio‐oncology, coronary artery disease, proportion, spectrum

## Abstract

**Background:**

Globally, coronary artery disease (CAD) and cancer are the leading causes of death. Studies focusing on the proportion and spectrum of cancer mortality among CAD patients are lacking. We aim to characterize the proportion and spectrum of cancer‐specific mortality among patients with CAD.

**Methods:**

We analyzed 93,797 hospitalized survivors with angiographically documented CAD between 2007 and 2020 (mean age: 62.8 ± 11.1 years, 24.7% female) from Cardiorenal ImprovemeNt II (CIN‐II) cohort.

**Results:**

During the median follow‐up of 4.8 years (IQR: 2.6–7.5), 13,162 (14.0%) patients died after discharge. A total of 1223/7703 (15.8% of cause‐specific death) CAD patients died of cancer. The three most common types of cancer‐specific death were lung (36.1%), liver (13.3%), and colorectum cancer (12.8%). Furthermore, male (adjusted HR 2.38, 95% CI: 1.99–2.85) and older (≥60 vs. <60 years, adjusted HR 3.25, 95%CI 2.72–3.88) patients had a significantly increased cancer‐specific mortality.

**Conclusions:**

Our data suggest that nearly one‐sixth of death is accounted for cancer among CAD patients within a median follow‐up of 4.8 years. Lung, liver, and colorectum cancer are top three cancer‐specific mortality. Further studies are needed to reduce cancer mortality for CAD patients, especially in older and male ones.

**Trail Registration:**

(ClinicalTrials.gov NCT05050877).

## 1 INTRODUCTION

Coronary artery disease (CAD) is the leading cause of death globally.^1^, ^2^ The prevalent case is 197, and 9.14 million patients die of CAD per year.^3^, ^4^ In China, there were up to 1.27 million increased incident CAD deaths from 1990 to 2019, accounting for 38.2% of the global incident of CAD mortality.^5^, ^6^

CAD survivors may have an increased risk of cancer either from shared lifestyles (tobacco smoking, obesity, processed meat diet, et al.) or experiencing same disease progression (inflammation, neurohormonal stress, angoigenesis, et al.).^7^, ^8^, ^9^, ^10^, ^11^ A US cohort study from 1991 to 2008 points out that 26.2% patients undergoing percutaneous coronary intervention (PCI) treatment die of cancer, which becomes the second main cause of death among patients undergoing PCI, and the cancer mortality shows an increasing trend over time in this study.^12^ Another US randomized control study has showed that the proportion of cancer mortality in all‐cause death is 25% among patients undergoing PCI treatment within 3‐year follow‐up.^13^ However, among patients with CAD, the proportion of cancer mortality is not well studied, especially under modern treatment of CAD and in developing country.

In addition, understanding the spectrum of extraordinary diversity of cancer will facilitate the development of cardio‐oncology management strategies. The 2020 Global Cancer Statistics have reported that lung, colorectum, and liver cancer remained the top 3 leading causes of cancer death globally.^14^ Nonetheless, rare studies focus on the spectrum of cancer‐specific mortality among CAD patients.

Based on a Chinese large‐scale cohort at contemporary management of CAD between 2007 and 2020, this study fills an important gap in the literature for incidence and spectrum of incident cancer mortality in CAD patients, which may prompt potential survival improvement in long‐term CAD and cancer management.

## 2 METHODS

### 2.1 Study population

**FIGURE 1:**
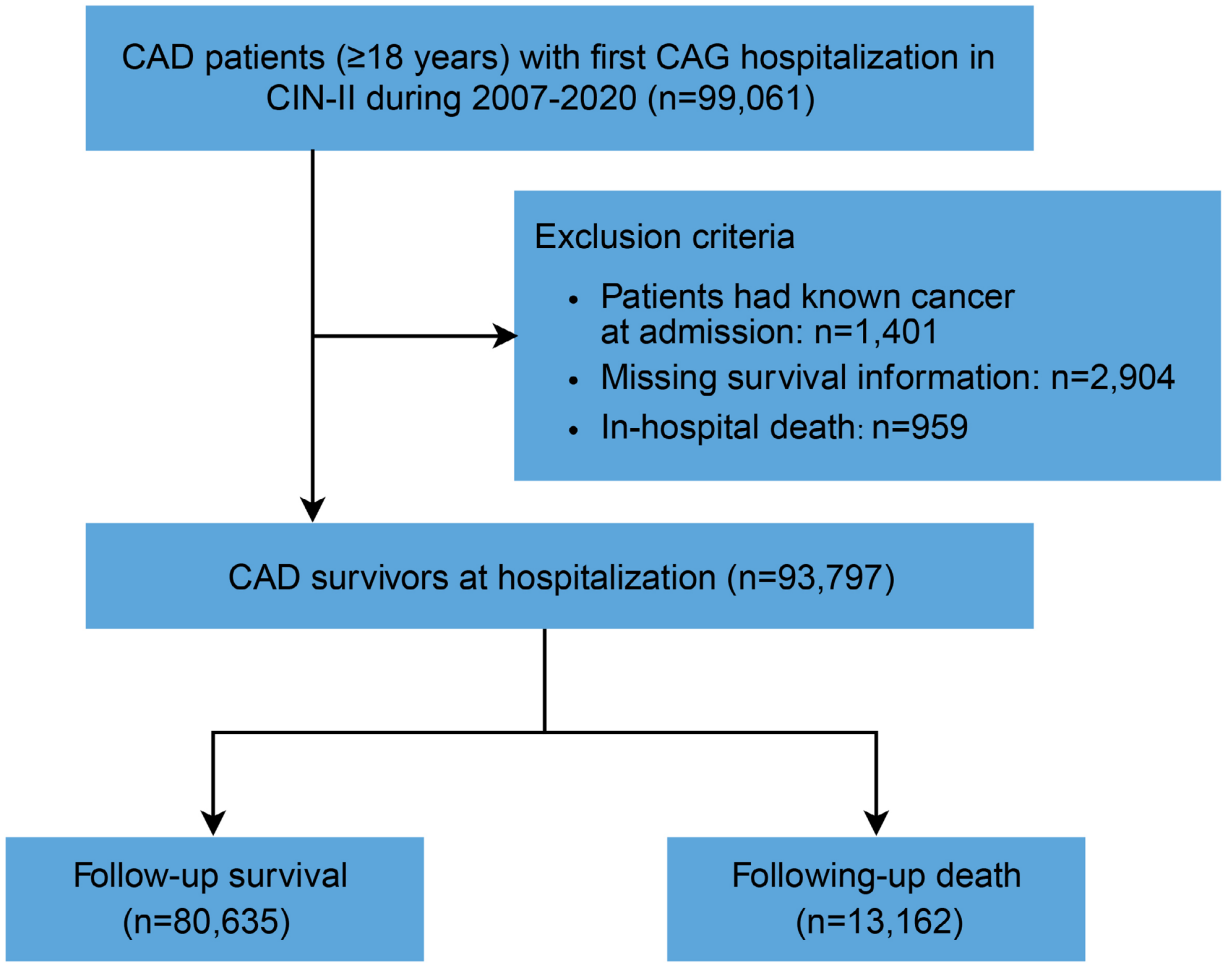
Flow diagram of study population selection. CAD, coronary artery disease; CAG, coronary angiogram.

This multi‐center, retrospective study was based on the coronary angiography (CAG) among Cardiorenal ImprovemeNt II cohort (CIN‐II, NCT05050877) among five regional central tertiary teaching hospitals in China.^15^ All consecutive CAD patients (≥18 years) firstly admitted for CAG were included from 2007 to 2020. Exclusion criteria are as follows: (1) Patients had known cancer at admission; (2) patients died during hospitalization; and (3) patients with missing survival information. Finally, 93,797 participants in CIN‐II were enrolled into study analysis (see Figure 1). The indication of CAG and/or PCI was according to the signs or symptoms of ischemia, diagnostic enzymes, or electrocardiogram. All patients' treatment performed in compliance with standard clinical guidelines.^16^, ^17^

The study protocol was approved by the Ethics Committee of Guangdong Provincial People's Hospital (No. GDREC2019‐555H‐2), all participating sites received institutional review board approval from their own ethics committees, and the study was performed according to the declaration of Helsinki.

### 2.2 Data collection

In‐hospital data were collected from the Electronic Clinical Management System, which was mainly included six sections: demographic, diagnostic information, laboratory indices, operations, medication, and discharge status. The details of data governance have been introduced in the previous study.^15^ To identify the survival information of patients with CAD, we also linked cause‐specific surveillance dataset at the regional Center for Disease Control and Prevention. Senior cardiologists were responsible for the data quality control and periodically carried out database checking.

### 2.3 Study outcomes and definitions

CAD was confirmed by coronary angiography, and the main artery stenosis was more than 30%; one of the other three coronary vessels stenosis was more than 50%.^15^ Chronic kidney disease was defined as an estimated glomerular filtration rate ≤ 60 mL/min/1.73 m^2^ using Chronic Kidney Diseases Epidemiology Collaboration equation.^18^ Anemia was defined as hematocrit <36% for female and <39% for male.^19^ Diabetes mellitus was assessed by the discharge diagnosis, or HbA1c >6.5%, or treatment with a hypoglycemic agent or insulin. Hyperlipemia was defined according to 2016 European Society of Cardiology guidelines.^20^ Cause‐specific mortality was categorized by the main reason of death with International Classification of Diseases (ICD) 10th Edition. Death case with missing values of ICD‐10 information was excluded from the case‐specific analysis. We classified total cancer mortality (ICD‐10: C00–C99, D01–D48) into lung cancer, liver cancer, colorectum cancer, and other cancer deaths (Table S1). The cardiovascular (CV) mortality was mainly identified by ICD‐10 codes: I00–I99, Q20–Q28, N00–N08, N10–N16, N17–N19.^21^

### 2.4 Statistical analysis

Proportion and spectrum of incident cancer‐specific mortality were used to further characterize the cancer burden among CAD patients without known cancer and identified death reason. We mainly characterized subjects into three groups among CAD patients: survival, cardiovascular death, and cancer death. Patients who died of cancer were further characterized the top 3 most common cancer for descriptive analysis. The categorical variables were presented as numbers (percentage), and continuous variables were presented as means (SD) or median [interquartile ranges (IQR)]. To compare the differences between survival, cardiovascular mortality, and cancer‐specific mortality, *t*‐test and standardized mean difference (SMD) have been used to assess parametric continuous variables, the Mann–Whitney *U* test for non‐parametric variables, the chi‐square test for categorical variables and Fisher's exact test for 2 × 2 tables.

We presented the proportion and spectrum of the mortality in the whole CAD patients and showed their development during the follow‐up period. Moreover, the difference of the proportion and spectrum of cancer‐specific mortality in sex and age groups (<60 and ≥60 years) were analyzed, respectively. To further assess the influence of sex and age in cancer‐specific mortality, Fine–Gray competing risk model was also conducted.^22^, ^23^ Clinically important variables were included in the final model, including demographic characteristics (age and sex), history of present illness (hypertension, diabetes, congestive heart failure, chronic kidney disease, atrial fibrillation, stroke, and anemia), laboratory examinations (low‐density lipoprotein cholesterol), hospital treatment, and medication information (PCI, angiotensin‐converting enzyme inhibitor or angiotensin receptor blocker [ACEI/ARB], β‐blocker, statins, and dual‐antiplatelet therapy). In addition, the hazard ratio (HR), 95% confidence interval (CI) and *p* value were calculated, and variables with a missing rate of more than 20% were excluded in multivariate analysis.

To further verify the reliability of the analysis, sensitivity analyses were conducted by fivefold predicted mean matching multiple interpolation (“VIM” package) to impute the missing data, which were enrolled in the final regression models.^24^ All tests in our study were two‐side analysis, and *p* value <0.05 was considered statistically significant. All analyses were performed using R version 4.1.2 (R Foundation for statistical computing).

## 3 RESULTS

### 3.1 Baseline characteristics

From 2007 to 2020, a total of 93,797 CAD patients without known cancer were enrolled in this study, of whom the mean age was 62.8 ± 11.1 years, and 23,143 (24.7%) patients were female (Table 1; Figure S1). Compared with survival patients, patients died of cancer were significantly older (cancer death vs. survival = 68.3 ± 8.7 vs. 62.1 ± 11.0, *p* value<0.001), but similar to cardiovascular death (*p* > 0.05). We also observed male with CAD were more likely to die of cancer than survival and cardiovascular death (cancer death vs. survival vs. cardiovascular death, 84.5% vs. 75.0% vs. 76.4%, *p* value<0.001). Meanwhile, compared with the survival, CAD patients who died of incident cancer were more likely to suffer from anemia and worsen kidney function, and ACEI/ARB, but with lower rate in AMI, PCI history, and β‐blockers medication (All *p* value <0.05). Other variables showed no difference, such as hypertension, diabetes mellitus, and congestive heart failure.

**TABLE 1:** Baseline characteristics of CAD patients based on the survival situation.

Characteristics	Overall	Cancer death^a^	Cardiovascular death^a^			Survival		
	*N* = 93,797	*N* = 1223	*N* = 5420	*p* value^b^	SMD^b^	*N* = 80,635	*p* value^c^	SMD^c^
Demographic characteristics								
Age (mean [SD])	62.8 (11.1)	68.3 (8.7)	67.9 (10.4)	0.295	0.035	62.1 (11.0)	<0.001	0.621
Female, *n* (%)	23,143 (24.7)	190 (15.5)	1279 (23.6)	<0.001	0.204	20,115 (24.9)	<0.001	0.236
Smoking history, *n* (%)	23,206 (36.2)	393 (41.3)	1657 (38.5)	0.114	0.058	19,627 (35.9)	0.001	0.111
Insurance, *n* (%)	79,538 (85.1)	1029 (84.1)	4638 (85.57)	0.217	0.040	68,797 (85.6)	0.151	0.042
Comorbidities								
Acute myocardial infarction, *n* (%)	26,095 (27.8)	289 (23.6)	1469 (27.1)	0.014	0.080	22,246 (27.6)	0.003	0.091
Hypertension, *n* (%)	51,966 (55.4)	679 (55.5)	3408 (62.9)	<0.001	0.150	44,211 (54.8)	0.651	0.014
Diabetes mellitus, *n* (%)	32,448 (34.6)	413 (33.8)	2515 (46.4)	<0.001	0.260	27,180 (33.7)	0.988	0.001
Congestive heart failure, *n* (%)	16,030 (17.1)	203 (16.6)	1660 (30.6)	<0.001	0.335	12,387 (15.4)	0.250	0.034
Chronic kidney disease, *n* (%)	17,865 (19.0)	319 (26.1)	2469 (45.6)	<0.001	0.415	13,195 (16.4)	<0.001	0.239
Atrial fibrillation, *n* (%)	4087 (4.4)	54 (4.4)	471 (8.7)	<0.001	0.173	3139 (3.9)	0.389	0.026
Stroke, *n* (%)	5899 (6.3)	74 (6.1)	569 (10.5)	<0.001	0.162	4737 (5.9)	0.843	0.007
Hyperlipemia, *n* (%)	53,585 (57.1)	714 (58.4)	3285 (60.6)	0.160	0.045	46,224 (57.3)	0.476	0.021
Anemia, *n* (%)	25,714 (31.0)	467 (41.4)	2565 (51.2)	<0.001	0.197	20,482 (28.7)	<0.001	0.269
Prior PCI, *n* (%)	9328 (9.9)	100 (8.2)	585 (10.8)	0.008	0.089	8147 (10.1)	0.030	0.067
Prior MI, *n* (%)	5832 (6.2)	79 (6.5)	492 (9.1)	0.004	0.098	4770 (5.9)	0.460	0.023
Prior CABG, *n* (%)	459 (0.5)	9 (0.7)	46 (0.8)	0.827	0.013	369 (0.5)	0.225	0.036
Laboratory tests								
eGFR, mL/min/1.73 m^2^	78.8 (26.0)	72.5 (21.0)	61.6 (28.8)	<0.001	0.433	80.7 (25.3)	<0.001	0.356
Hemoglobin, g/L	134.3 (17.6)	131.0 (18.0)	125.4 (20.7)	<0.001	0.286	135.2 (17.0)	<0.001	0.243
Preoperative SCr, μmol/L	1.0 [0.8, 1.1]	1.0 [0.8, 1.2]	1.1 [0.9, 1.5]	<0.001	0.385	0.9 [0.8, 1.1]	<0.001	0.059
LDLC, mmol/L	2.9 (1.0)	2.6 (0.9)	2.8 (1.0)	<0.001	0.166	2.9 (1.0)	<0.001	0.308
HDLC, mmol/L	1.0 (0.3)	1.0 (0.3)	1.0 (0.3)	0.585	0.019	1.0 (0.3)	<0.001	0.208
hs‐TnT, ng/L	14.4 [7.7, 68.7]	20.1 [10.4, 139.6]	54.5 [20.7, 407.0]	<0.001	0.235	13.2 [7.5, 53.3]	<0.001	0.002
NT‐proBNP, pg/mL	262.0 [70.0, 1097.0]	349.6 [88.4, 1287.2]	1523.0 [410.0, 4470.0]	<0.001	0.508	212.2 [62.9, 856.0]	<0.001	0.122
LVEF, *n* (%)	59.0 (11.8)	58.8 (11.7)	51.0 (14.8)	<0.001	0.585	59.8 (11.2)	0.027	0.081
Procedures								
PCI, *n* (%)	67,501 (72.0)	912 (74.6)	3897 (71.9)	0.064	0.060	58,008 (71.9)	0.045	0.059
Bare metal stent, *n* (%)	2165 (2.3)	48 (3.9)	174 (3.2)	0.243	0.039	1613 (2.0)	<0.001	0.114
Drug‐eluting stents, *n* (%)	64,162 (68.4)	854 (69.8)	3632 (67.0)	0.062	0.061	55,328 (68.6)	0.381	0.026
CABG, *n* (%)	124 (0.1)	1 (0.1)	15 (0.3)	0.350	0.046	96 (0.1)	>0.999	0.012
Discharge medication								
Dual‐antiplatelet therapy, *n* (%)	71,866 (80.7)	949 (79.9)	4004 (79.3)	0.623	0.017	61,927 (80.7)	0.527	0.019
ACEI/ARB, *n* (%)	62,643 (70.4)	864 (72.8)	3664 (72.5)	0.884	0.006	53,692 (70.0)	0.040	0.062
β‐blocker, *n* (%)	71,388 (80.2)	925 (77.9)	4059 (80.3)	0.067	0.060	61,618 (80.3)	0.044	0.059
Statins, *n* (%)	84,570 (95.0)	1134 (95.5)	4717 (93.4)	0.007	0.095	72,978 (95.1)	0.562	0.019

Abbreviations: ACEI/ARB, Angiotensin‐converting enzyme inhibitors or angiotensin receptor blocker; CABG, coronary artery bypass grafting; eGFR, estimated glomerular filtration rate epidemiology collaboration equation; HDLC, high density lipoprotein cholesterol; hs‐TnT, high sensitivity Troponin T; LDLC, low‐density lipoprotein cholesterol; LVEF, left ventricular ejection fraction; NT‐proBNP, N‐terminal pro‐B‐type natriuretic peptide; PCI, percutaneous coronary intervention; prior CABG, prior coronary artery bypass grafting; prior MI, prior myocardial infarction; prior PCI, prior percutaneous coronary intervention; SCr, serum creatinine.

^a^ In patients who were identified of the cause‐specific death.

^b^ Cancer death vs. cardiovascular death.

^c^ Cancer death vs. survival.

### 3.2 Proportion and spectrum of cancer‐cause mortality

**FIGURE 2:**
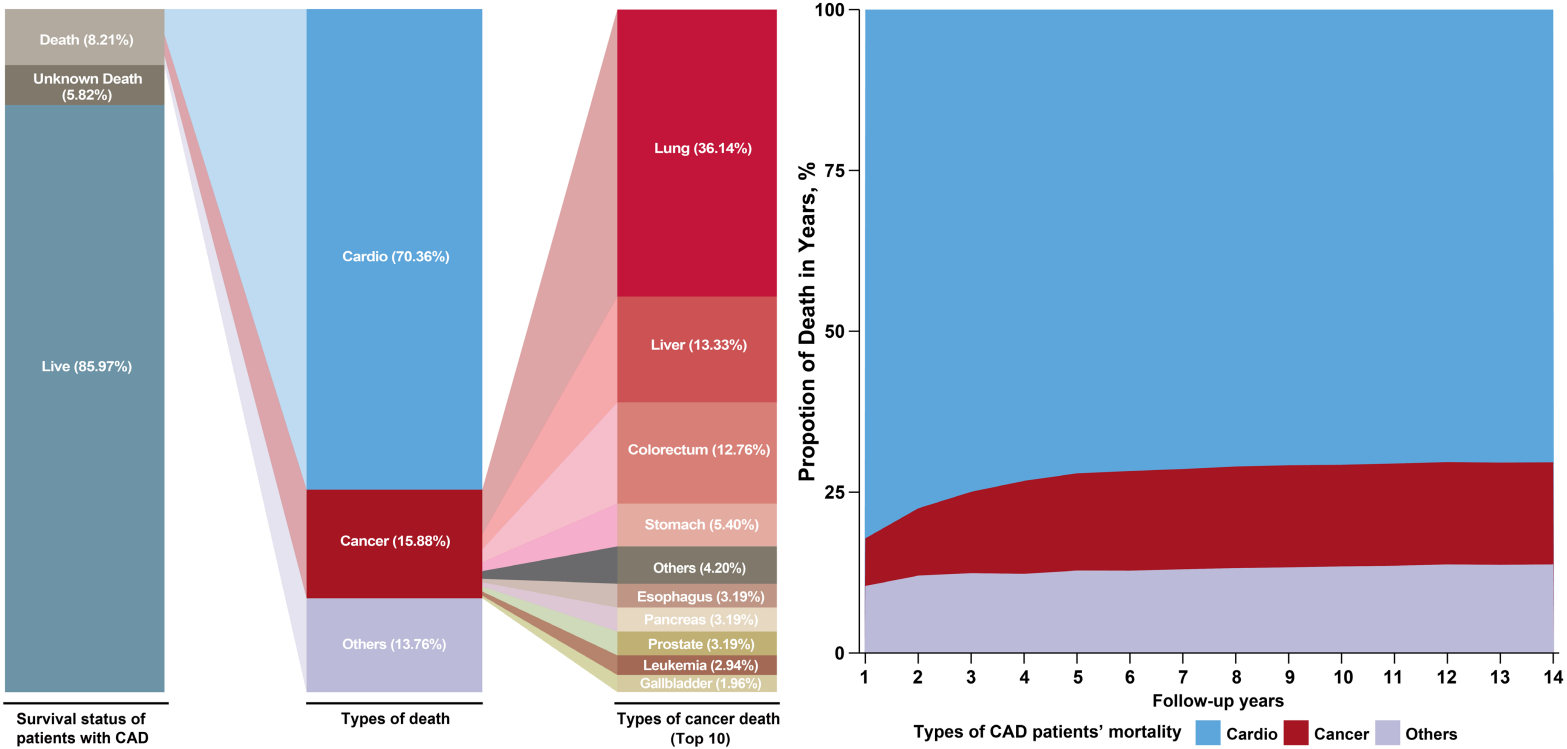
The cumulative proportion and development of cause‐specific mortality in patients with CAD. CAD, coronary artery disease.

Within the median follow‐up of 4.8 years (IQR 2.6–7.5), in 93,797 CAD patients, 14.0% CAD patients died (*n* = 13,162). The majority death 70.4% (5420/7703) was due to cardiovascular diseases, followed by cancer 15.9% (1223/7703) among patients who had identified death cause (Figures 1 and 2). During the follow‐up, the proportion of cancer deaths increased over time (Figure 2), and the top three specific death of cancer were lung 36.1% (442/1223), liver 13.3% (163/1223), and colorectum cancer 12.8% (156/1223) (Tables S2 and S3).

### 3.3 Proportion and spectrum of cancer‐cause mortality regarding age and sex differences

**FIGURE 3:**
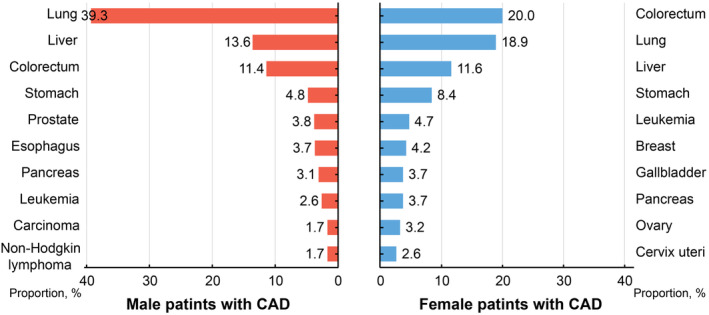
The top 10 most common of cancer‐specific mortality among CAD patients in sex groups. CAD, coronary artery disease.

**FIGURE 4:**
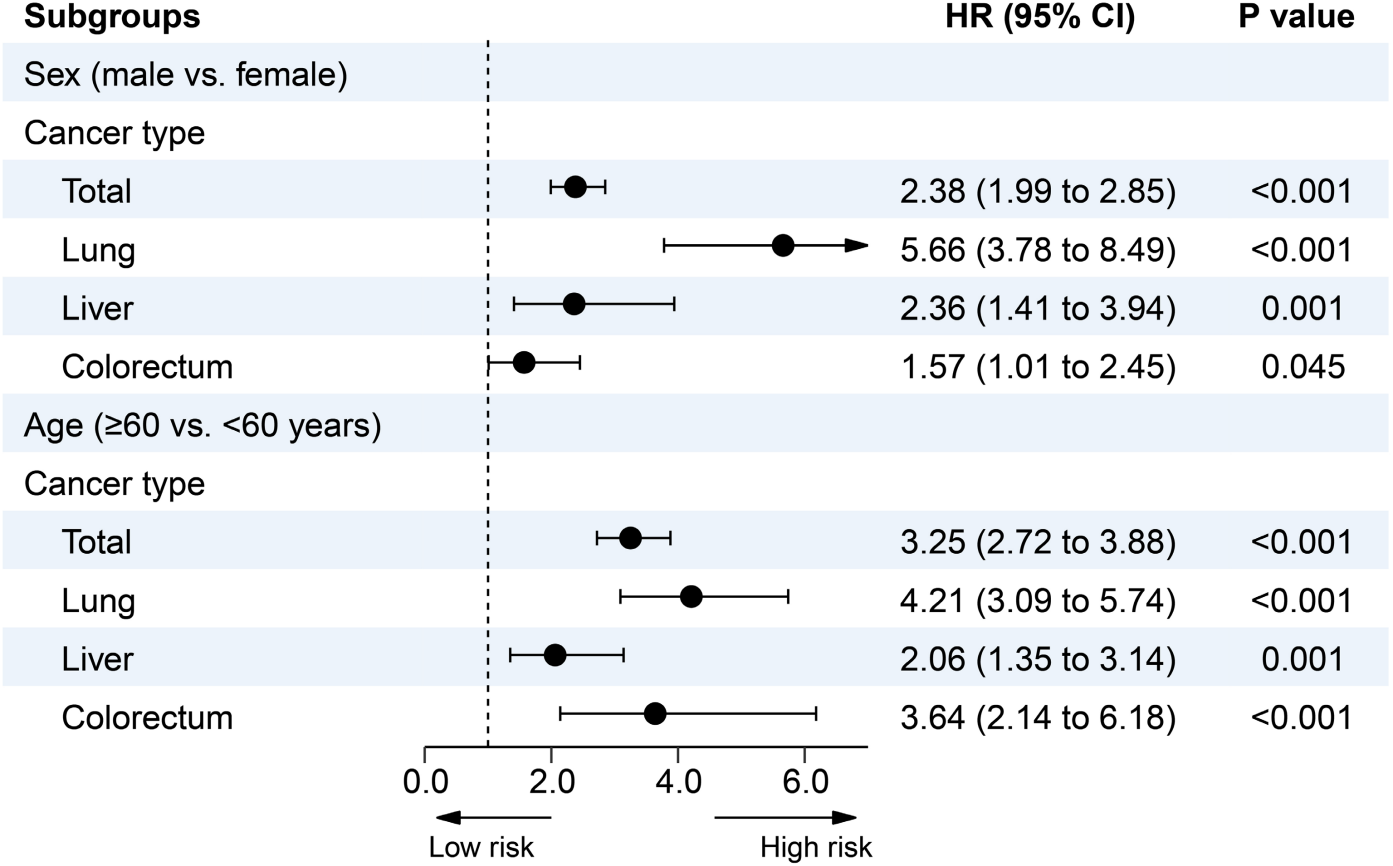
Hazard ratios and 95% confidence intervals of cancer‐specific mortality in age and sex groups. Fine–Gray competing risk model was adjusted for age, sex, hypertension, diabetes, congestive heart failure, chronic kidney disease, atrial fibrillation, stroke and anemia, low‐density lipoprotein cholesterol, percutaneous coronary intervention, angiotensin‐converting enzyme inhibitor or angiotensin receptor blocker, β‐blocker, statins, and dual‐antiplatelet therapy.

Of 1223 CAD patients dying of cancer, 190 (15.5%) were females. The sex different analysis in spectrum of cancer‐specific mortality showed the three most common cancer mortality in male were lung, liver, and colorectum cancer, but for female, they were changed into colorectum, lung, and liver cancer (Figure 3; Table S4). Compared to female patients with CAD, male patients had a higher risk of long‐term cancer mortality among patients with identified cause‐specific death (adjusted HR 2.38; 95CI% 1.99 to 2.85, *p* < 0.001, see in Figure 4).

In addition, compared with patients <60 years, male ≥60 years had a higher risk of cancer death, including lung, liver, and colorectum cancer; female patients ≥60 years also had a higher risk of cancer death, but there was no significant difference in the risk of lung, liver, and colorectum cancer in females. After imputing the missing value, similar results were still observed in sensitivity analysis (Figures S2–S4).

## 4 DISCUSSION

In this large‐scale contemporary cohort study, it is the first time to systematically analyze the proportion and spectrum of cancer mortality in patients with CAD. Within the prolonged follow‐up period, the proportion of cancer deaths increase over time, with one‐sixth of CAD patients dying of cancer. In patients with CAD, the top three cancer deaths are lung, liver, and colorectum cancer, and the spectrum of cancer mortality in males is similar to the overall CAD patients. However, it changes to colorectum, lung, and liver cancer mortality in female patients. Male and older patients have onefold to twofold increased risk of cancer mortality.

There are many controversies in clinical treatment between CAD and cancer, but most of studies have been conducted in cancer patients to estimate the incidence and mortality of CAD, and few studies indicate the proportion and spectrum of incident cancer in CAD patients.^25^, ^26^ A 6.5 million US National Inpatients Sample database reported that nearly 10% subjects have current or historical cancer among patients with AMI at admission (top 4 prevalent cancer such as lung, prostate, breast, and colon cancer), and a concomitant cancer diagnosis is associated with higher risk of in‐hospital mortality.^12^ However, the lack of post‐discharge cancer mortality information poses limitations on the implementation of effective cancer prevention and treatment strategies for CAD patients. Rajiv Gulati et al. have demonstrate that the 5‐year cancer mortality for patients undergoing PCI treatment is up to 26.2%.^2^ Meanwhile, DAPT study mainly enrolls patients with dual‐antiplatelet therapy after coronary stents, and nearly 25% patients die of cancer‐related reason among 3‐year follow‐up period.^13^ The proportion of cancer‐relative death among the two studies are higher than 15% in our study, and this difference may mainly account for the different category of CAD patients: Patients with AMI or severe lesions may have a higher inflammation level, which may increase the risk of incident cancer.^27^ In addition, cancer screening is not widely conducted in China, and it may lead to underestimation of cancer mortality. The TRILOGY ACS study demonstrates a similar risk of developing cancer during treatment with Prasugrel vs. Clopidogrel (plus aspirin), and a total of 1.8% (160/9105) patients occurred non‐benign neoplasm during median 15 month follow‐up period.^28^ Although this study shows a list of incident cancer, due to the limitation of sample size and follow‐up duration, it still cannot describe the spectrum of incident cancer among CAD patients, especially the spectrum of incident cancer‐specific death among these patients, but it enrolls a large number of CAD patients with or without acute coronary syndromes, which may be more necessary to reflect the burden of cancer mortality in CAD patients.

Furthermore, 2020 Global Cancer Statistics has reported lung cancer remained the leading cause of cancer death (18.0%), followed by colorectal (9.4%), liver (8.3%), and stomach cancer (7.7%). Males have the similar proportion with cancer‐specific death among overall population, but females would change into breast (15.5%), lung (13.7%), colorectum (9.5%), and cervix uteri (7.7%).^14^ Our study indicated that the proportion and spectrum of cancer mortality among patients with CAD (lung [36.1%], liver [13.3%], and colorectum [12.8%], etc.) is different from the 2020 Global Cancer Statistics report, which may be caused by smoking, air pollution (lung cancer), chronic hepatitis B virus infection, aflatoxin exposure, non‐alcoholic fatty liver disease (liver cancer), heavy alcohol consumption, and consumption of red or processed meat (colorectum cancer). In addition, in the diagnosis and treatment for CAD patients, radiation exposure is inevitable and may increase the risk of carcinogenesis (like lung cancer), especially in patients who have received multiple CAG.^29^ Genetic components have also been proved to contribute to cancer susceptibility.^30^

For the proportion of cancer mortality by sex, we notice a significantly higher incidence of lung cancer mortality in male patients (39.3%) compared to female patients (18.9%). This disparity may be attributed to the prevalence of common risk factors for cardio‐oncology diseases in males, such as smoking, which induces the alterations in lipid metabolism, and lead to atherosclerosis and plaque progression.^31^, ^32^, ^33^ Alcohol consumption, obesity, and low consumption of fruit and vegetable, etc., also contribute to the high cancer mortality to a certain extent.^7^, ^11^ Furthermore, the three most common cancer mortality in female patients with CAD is colorectum (20.0%), lung (18.9%), and liver (11.6%) cancer in our study, which is different from the report (female breast, lung, and colorectum cancer). The high colorectum cancer mortality may mainly account for aging and antiplatelet therapy in CAD patients, which leads to the high risk of bleeding (fecal occult blood test positive) and further increases the diagnosis rate of cancer.

To date, the potential CV toxicities of cancer therapy are now widely recognized,^34^, ^35^ but the potential mechanisms underlying the relationship between CAD and cancer remain poorly understood. Biological systems are constantly exposed to oxidants, either from endogenous metabolic reactions or exogenous sources (e.g., toxins and smoking), and oxidative stress results from an imbalance of oxidant and antioxidant substances, a potential pathogen in tumorigenesis for enhancing oxidative activity in tumor tissues.^36^ Oxidative stress is associated with chronic inflammation (e.g., diabetes mellitus, hypertension, and obesity), which is commonly occurred in subjects with both CVD and cancer.^37^ What's more, lipid metabolism disorder commonly occurs in cancer cells, with the fatty acid chain becoming longer and saturated, leading to the inability to resist apoptosis.^38^ The increased production of isoprene from acetyl‐coenzyme A is also associated with cholesterol biosynthesis.^39^ The abnormal lipidomics profiles in cancer cells result in the alternation of breath volatile organic compounds, which shows that most volatile organic compounds are consistent with broad spectrum cancers.^40^

The present study indicates the increased competing risk from cancer‐specific mortality. The 2019 ESC Guidelines of chronic coronary syndromes management has suggested that the follow‐up management of CAD patients should not only focus on the prevention and control of CVD risk factors, but also pay attention to patients with high risk of cancer and cancer survivor, which may deserve more intensive risk screening, counseling, and management.^41^, ^42^, ^43^, ^44^ However, there is still less evidence on the proportion and spectrum of incident cancer mortality among patients with CAD. In addition, elder and male patients with CAD have remarkably increased risk of cancer mortality. Meanwhile, the proportion and spectrum of death for male and female patients with CAD are significantly different from the overall population, which suggests that we should make adjustments to the cancer prevention and treatment for those patients. Early cancer screen (pulmonary computerized tomography, volatile organic compounds test, gastrointestinal endoscopy, biomarker, et al.) and multidisciplinary care team organization are necessary for cardio‐oncology management.^8^, ^26^, ^40^ Risk factor control and lifestyle management (smoking cessation, weight reduction, daily exercise, balanced diet, etc.) will also help to improve the prognosis of patients with CAD. Further studies may need to explore the accurate prediction tool of cancer by clinical multiomics information, and further investigate the underlying mechanisms linking CAD and cancer mortality, which may help clinicians identify the high risk of cancer earlier and provide valuable insights for prevention and treatment.

This study still has several limitations. Firstly, this study has limitations inherent from retrospective analysis, including data incompleteness, without the follow‐up information of cancer‐related incidence and treatment. However, this study is a large‐scale longitudinal cohort study among patients undergoing CAG, and linking to multiple cause‐specific mortality dataset, which can provide the evidence of natural disease burden of cancer in these patients. Secondly, the potential for residual confounding cannot be excluded given the observational nature of this study such as traditional risk factors for cardio‐oncology, occupational, and screening for cancer (lung computerized tomography, gastroenteroscopy, cancer‐relative biomarkers, et al), but we adopted the data filling method to test the stability of the results in the multi‐factor correction of major problems. More prospective studies are needed in elderly patients with CAD for cancer screening to improve the treatment rate of cardio‐oncology disease.

## 5 CONCLUSION

Nearly one‐sixth of CAD patients died of cancer among the nearly 5‐year follow‐up. Lung, liver, and colorectum cancer are three most common types of cancer death in patients with CAD. It is necessary to carry out the cancer screening for patients with CAD in clinical practices, especially in elder and male patients.

## AUTHOR CONTRIBUTIONS

**Jin Liu:** Data curation (lead); formal analysis (lead); methodology (equal); supervision (lead); writing – original draft (lead); writing – review and editing (lead). **Shiqun Chen:** Data curation (equal); investigation (equal); methodology (equal); supervision (equal). **Yang Zhou:** Visualization (equal); writing – original draft (equal); writing – review and editing (equal). **Haozhang Huang:** Investigation (equal); resources (equal). **Qiang Li:** Investigation (equal); resources (equal). **Yan Liang:** Investigation (equal); resources (equal). **Shaohong Dong:** Investigation (equal); resources (equal). **Xiaoyu Huang:** Investigation (equal); resources (equal). **Liling Chen:** Investigation (equal); resources (equal). **Xueyan Zheng:** Investigation (equal); resources (equal). **Ruilin Meng:** Investigation (equal); resources (equal). **Congzhuo Jia:** Writing – review and editing (equal). **Jiyan Chen:** Funding acquisition (equal); project administration (equal); supervision (equal). **Ning Tan:** Funding acquisition (equal); project administration (equal); supervision (equal). **Yong Liu:** Funding acquisition (equal); investigation (equal); project administration (equal); supervision (equal).

## FUNDING INFORMATION

This work was supported by grants from Guangdong Provincial science and technology project (2020B1111170011), Guangdong Provincial science and technology project (KJ022021049), and Guangdong Provincial Key Laboratory of Coronary Heart Disease Prevention (No. Y0120220151). The work was not funded by any industry sponsors.

## CONFLICT OF INTEREST STATEMENT

The authors declare that they have no relevant financial interests.

## INFORMED CONSENT STATEMENT

Informed consent was obtained from all subjects involved in the study.

## Supporting information


Data S1

## Data Availability

Research data are stored in an institutional repository and will be shared upon request to the corresponding author.
