# Supplementary material for: Proportion and number of incident cancer deaths in coronary artery disease
Source: Cancer Med. 2023 Sep 27;12(19):20140–9. doi: 10.1002/cam4.6595 (PMC10587929; doi:10.1002/cam4.6595)
Supplement: Supplementary file 1 — Data S1 [file CAM4-12-20140-s001.docx]

**Supplementary**

Definitions

CHF was confirmed by the 10th Revision Codes of the International Classification of Diseases (ICD-10) for congestive heart failure (I50.001, I11.000, I13.000, I50.907 and R57.000 etc) and the heart function classification of the New York Heart Association (NYHA) class > 2 or Killip class > 1. CKD was defined as Estimated glomerular filtration rate (eGFR) < 60 mL/min/1.73m^2^. eGFR was calculated by the Chronic Kidney Diseases Epidemiology Collaboration equation (CKD-EPI): 1) Male and Scr ≤ 0.9 mg/dL, eGFR = 141×(Scr / 0.9)^-0.411^×0.993^Age^; 2) Male and Scr > 0.9 mg/dL, eGFR = 141×(Scr / 0.9)^-1.2091^×0.993^Age^; 3) Female and Scr ≤ 0.9 mg/dL, eGFR = 144×(Scr / 0.9)^-0.329^×0.993^Age^; 4)Female and Scr > 0.9 mg/dL, eGFR = 144×(Scr / 0.9)^-1.209^×0.993^Age^. ^$^ Diabetes mellitus (DM) was defined as ICD-10 codes, the utilization of oral hypoglycemic drugs or insulin when discharged from the hospital or the saccharification more than 6.5%.

$: Huber M, Ozrazgat-Baslanti T, Thottakkara P, Scali S, Bihorac A, Hobson C. Cardiovascular-Specific Mortality and Kidney Disease in Patients Undergoing Vascular Surgery. *JAMA Surg* 2016; 151(5): 441-50.

**Supplemental Table 1** The count of cancer-specific mortality among patients with coronary artery disease.

| **Cancer type** |  | **Number** |
| --- | --- | --- |
| Lung cancer | C33 C34 D38.1 C39 C78.0 D02.1 D02.2 D02.3 D02.4 | 443 |
| Liver cancer | C22 C22.9 C22.0 C22.1 C22.7 C22.3 C22.4 | 163 |
| Colorectum cancer | C18.9 C20 C18.7 C18 C18.2 C18.4 C18.6 C26.0 C17.0 C19 C78.5 C18.3 C17.9 C18.0 C17 C21.8 C17.2 D01.0 C18.8 C78.4 D01.2 D01.4 C17.1 C17.8 C21.0 | 156 |
| Stomach cancer | C16.9 C16 C16.8 D00.2 C16.1 C16.2 C16.5 C16.6 | 66 |
| Esophagus cancer | C15.9 C15 C15.4 C15.0 C15.1 C15.5 C15.3 C15.8 C15.2 D00.1 | 39 |
| Pancreas cancer | C25.9 C25.4 C25.0 C25 C25.1 C25.2 C25.7 C25.8 | 39 |
| Prostate cancer | C61 D07.5 | 39 |
| Leukemia | C92.0 C95.9 C95.0 C91.0 C95 C92.7 C93.0 C91.1 C92.4 C91.9 C92.1 C95.1 C92.5 C90.1 C93.1 C94.7 C91.5 C91 C94.2 C91.7 C92 C92.9 C93.2 C91.38 | 36 |
| Nervous cancer | C70 C71 C71.0 C71.5 C71.6 C71.9 C72 C72.5 C72.8 C72.9 C79.3 C47.9 C47 C47.4 C48.2 C79.4 D32 D42.0 D32.9 D43 D43.1 D33.3 D33.0 D33.7 C75.2 | 20 |
| Non-Hodgkin lymphoma | B21.2 C82 C83 C85 C84 | 20 |
| Multiple myeloma | C90.0 | 15 |
| Breast cancer | C50.9 C50 C50.8 D05.7 C50.2 D05 D05.9 C50.1 | 9 |
| Bone cancer | C13 C32.3 C40 C41 C48 D16 D48 | 6 |
| Ovaries cancer | C56 C79.6 D27 D39 | 6 |
| Thyroid cancer | C73 D34 | 6 |
| Cervical cancer | C53.9 C53 D06 C53 | 5 |
| Uterine cancer | C54 C55 C57.3 C57.4 D07.0 | 4 |
| Melanoma cancer | C43 C43.4 C43.4 C43.9 | 3 |
| Others |  | 148 |

**Supplemental Table 2** Baseline characteristics of top 3 most common types for cancer-specific death among CADs.

| **Characteristics** | **Overall** | **Lung** | **Liver** | **Colorectum** |
| --- | --- | --- | --- | --- |
|  | **93,797** | **442** | **163** | **156** |
| **Demographic characteristics** | | | | |
| Age (mean (SD)) | 62.8 (11.1) | 68.3 (8.1) | 66.8 (9.6) | 69.1 (9.2) |
| Female, n(%) | 23143 (24.7) | 36 (8.1) | 22 (13.5) | 38 (24.4) |
| Smoking history, n(%) | 23206 (36.2) | 178 (55.8) | 54 (39.7) | 37 (29.1) |
| Insurance, n(%) | 79538 (85.1) | 366 (82.8) | 143 (87.7) | 131 (84.0) |
| **Comorbidities** | | | | |
| Acute myocardial infarction, n(%) | 26095 (27.8) | 126 (28.5) | 36 (22.1) | 32 (20.5) |
| Hypertension, n(%) | 51966 (55.4) | 222 (50.2) | 87 (53.4) | 92 (59.0) |
| Diabetes mellitus, n(%) | 32448 (34.6) | 120 (27.1) | 58 (35.6) | 67 (42.9) |
| Congestive heart failure, n(%) | 16030 (17.1) | 86 (19.5) | 24 (14.7) | 27 (17.3) |
| Chronic kidney disease, n(%) | 17865 (19.0) | 104 (23.5) | 40 (24.5) | 46 (29.5) |
| Atrial fibrillation, n(%) | 4087 (4.4) | 18 (4.1) | 4 (2.5) | 4 (2.6) |
| Stroke, n(%) | 5899 (6.3) | 31 (7.0) | 7 (4.3) | 8 (5.1) |
| Hyperlipemia, n(%) | 53585 (57.1) | 252 (57.0) | 93 (57.1) | 91 (58.3) |
| Anemia, n(%) | 25714 (31.0) | 178 (44.4) | 49 (32.5) | 61 (41.8) |
| Prior PCI, n(%) | 9328 (9.9) | 38 (8.6) | 10 (6.1) | 11 (7.1) |
| Prior MI, n(%) | 5832 (6.2) | 38 (8.6) | 8 (4.9) | 5 (3.2) |
| Prior CABG, n(%) | 459 (0.5) | 2 (0.5) | 2 (1.2) | 1 (0.6) |
| **Laboratory tests** | | | | |
| eGFR, mL/min/1.73m2 | 78.8 (26.0) | 72.8 (20.0) | 74.2 (21.3) | 70.5 (20.5) |
| Hemoglobin, g/L | 134.3 (17.6) | 131.7 (17.4) | 134.0 (17.2) | 130.4 (18.2) |
| Preoperative SCr, umol/L | 1.0 [0.8, 1.1] | 1.0 [0.9, 1.2] | 1.0 [0.9, 1.2] | 1.0 [0.8, 1.2] |
| LDLC, mmol/L | 2.9 (1.0) | 2.7 (0.9) | 2.6 (0.9) | 2.6 (0.9) |
| HDLC, mmol/L | 1.0 (0.3) | 1.0 (0.2) | 1.0 (0.3) | 1.0 (0.3) |
| hs-TnT, ng/L | 14.4  [7.7, 68.7] | 23.1  [11.0, 237.2] | 22.0  [11.1, 185.9] | 20.0  [10.8, 93.1] |
| NT-proBNP, pg/mL | 262.0  [70.0, 1097.0] | 398.1  [113.3, 1199.5] | 516.0  [81.3, 1478.0] | 341.7  [111.6, 1510.5] |
| LVEF, n(%) | 59.0 (11.8) | 59.5 (11.4) | 57.3 (13.2) | 59.3 (10.2) |
| **Procedures** | | | | |
| PCI, n(%) | 67501 (72.0) | 339 (76.7) | 119 (73.0) | 121 (77.6) |
| Bare metal stent, n(%) | 64162 (68.4) | 312 (70.6) | 114 (69.9) | 110 (70.5) |
| Drug-eluting stents, n(%) | 2165 (2.3) | 23 (5.2) | 3 (1.8) | 9 (5.8) |
| CABG, n(%) | 124 (0.1) | 1 (0.2) | 0 (0.0) | 0 (0.0) |
| **Discharge medication** | | | | |
| Dual-antiplatelet therapy, n(%) | 71866 (80.7) | 353 (82.5) | 125 (77.6) | 120 (77.9) |
| ACEI/ARB, n(%) | 71388 (80.2) | 331 (77.3) | 124 (77.0) | 123 (79.9) |
| β-blocker, n(%) | 84570 (95.0) | 415 (97.0) | 150 (93.2) | 147 (95.5) |
| Statins, n(%) | 86795 (97.5) | 419 (97.9) | 155 (96.3) | 151 (98.1) |

Abbreviation: ACEI/ARB: Angiotensin converting enzyme inhibitors or angiotensin receptor blocker; CABG: Coronary artery bypass grafting; eGFR: Estimated glomerular filtration rate epidemiology collaboration equation; HDLC: High density lipoprotein cholesterol; hs-TnT: High sensitivity Troponin T; LDLC: Low density lipoprotein cholesterol; LVEF: Left ventricular ejection fraction; NT-proBNP: N-terminal pro-B-type natriuretic peptide; PCI: Percutaneous coronary intervention; Prior PCI: Prior percutaneous coronary intervention; Prior MI: Prior myocardial infarction; Prior CABG: Prior coronary artery bypass grafting; SCr: serum creatinine.

**Supplemental Table 3** Distribution of top 10 most common types for cancer-specific death: global data vs. our cohort.^#^

| **Worldwide** | | **Our cohort** | |
| --- | --- | --- | --- |
| **Cancer type** | **Proportion** | **Cancer type** | **Proportion** |
| Lung | 18.0% | Lung | 36.1% |
| Colorectum | 9.4% | Liver | 13.3% |
| Liver | 8.3% | Colorectum | 12.8% |
| Stomach | 7.7% | Stomach | 5.4% |
| Female breast | 6.9% | Esophagus | 3.2% |
| Esophagus | 5.5% | Pancreas | 3.2% |
| Pancreas | 4.7% | Prostate | 3.2% |
| Prostate | 3.8% | Leukemia | 2.9% |
| Cervix uteri | 3.4% | Gallbladder | 2.0% |
| Leukemia | 3.1% | Carcinoma | 1.8% |

**#:** Sung H, Ferlay J, Siegel RL, et al. Global Cancer Statistics 2020: GLOBOCAN Estimates of Incidence and Mortality Worldwide for 36 Cancers in 185 Countries. *CA Cancer J Clin* 2021; 71(3): 209-49.

**Supplemental Table 4** Distribution of top 10 most common types for cancer-specific death in male and female: global data vs. our cohort.^#^

| **Sex** | **Worldwide** | | **Our cohort** | |
| --- | --- | --- | --- | --- |
|  | **Cancer type** | **Proportion** | **Cancer type** | **Proportion** |
| **Male** | Lung | 21.5% | Lung | 39.3% |
|  | Liver | 10.5% | Liver | 13.6% |
|  | Colorectum | 9.3% | Colorectum | 11.4% |
|  | Stomach | 9.1% | Stomach | 4.8% |
|  | Prostate | 6.8% | Prostate | 3.8% |
|  | Esophagus | 6.8% | Esophagus | 3.7% |
|  | Pancreas | 4.5% | Pancreas | 3.1% |
|  | Leukemia | 3.2% | Leukemia | 2.6% |
|  | Bladder | 2.9% | Carcinoma | 1.7% |
|  | Non-hodgkin lymphoma | 2.7% | Non-hodgkin lymphoma | 1.7% |
| **Female** | Female breast | 15.5% | Colorectum | 20.0% |
|  | Lung | 13.7% | Lung | 18.9% |
|  | Colorectum | 9.5% | Liver | 11.6% |
|  | Cervix uteri | 7.7% | Stomach | 8.4% |
|  | Stomach | 6.0% | Leukemia | 4.7% |
|  | Liver | 5.7% | Female breast | 4.2% |
|  | Pancreas | 4.9% | Gallbladder | 3.7% |
|  | Ovary | 4.7% | Pancreas | 3.7% |
|  | Esophagus | 3.8% | Ovary | 3.2% |
|  | Leukemia | 3.0% | Cervix uteri | 2.6% |

**#:** Sung H, Ferlay J, Siegel RL, et al. Global Cancer Statistics 2020: GLOBOCAN Estimates of Incidence and Mortality Worldwide for 36 Cancers in 185 Countries. *CA Cancer J Clin* 2021; 71(3): 209-49.


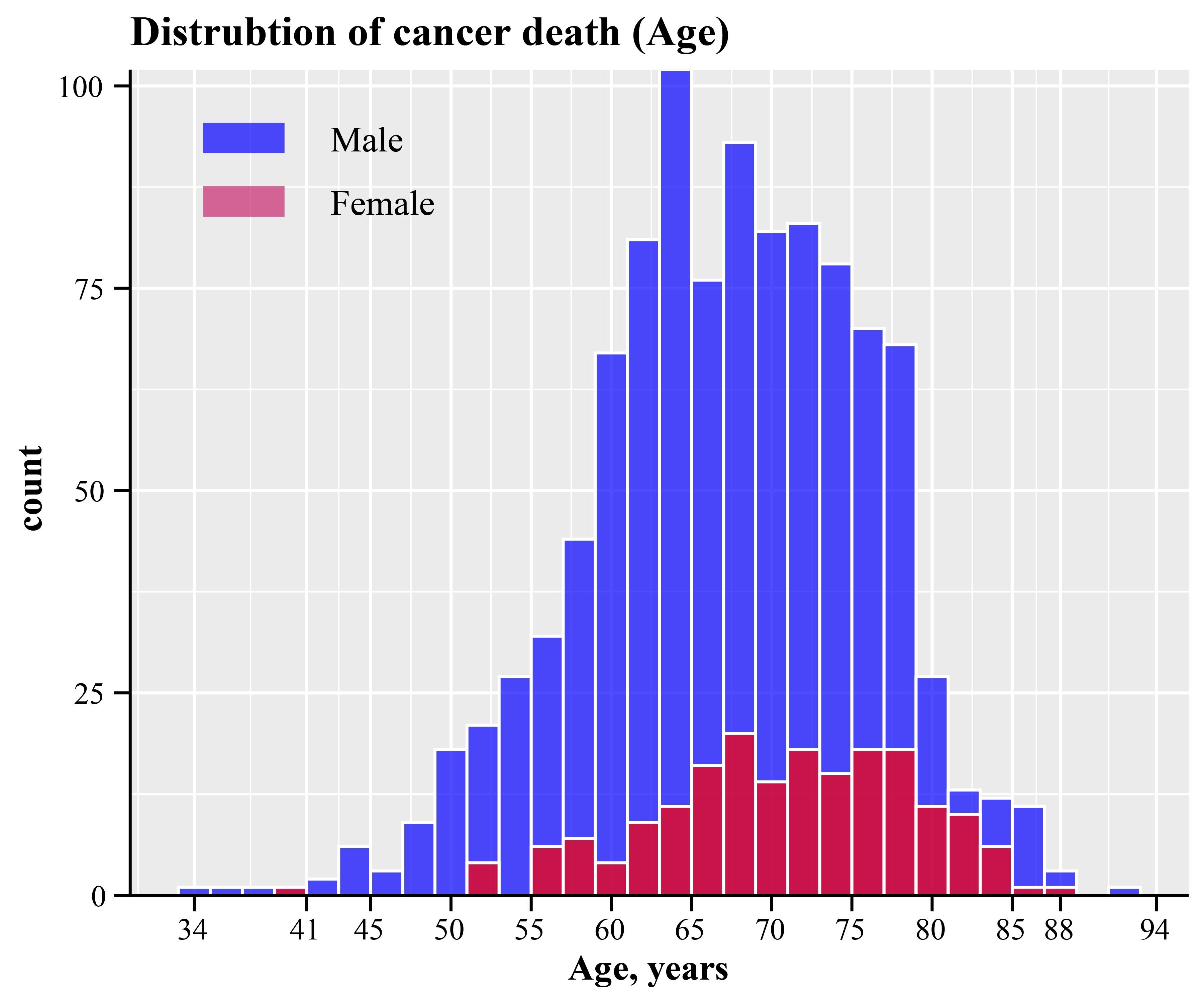


**Supplemental Figure 1** The age distribution of cancer mortality stratified by sex.


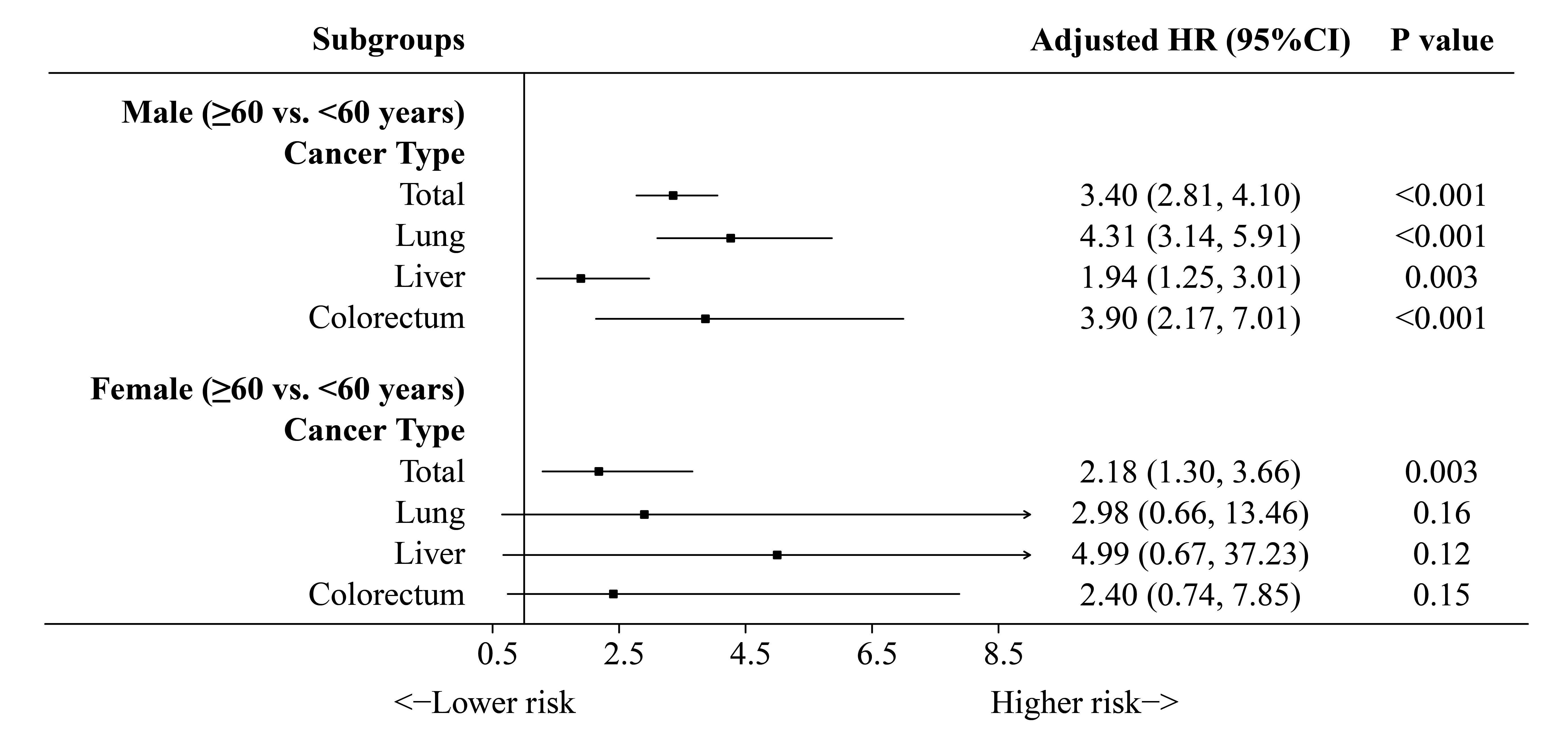
**Supplemental Figure 2** Hazard ratios (HR) and 95% confidence intervals (CIs) of cancer-specific mortality in sex and different age groups

**P* for interaction test: multiplicative interaction analysis between sex and different age groups (<60 and ≥60 years).
Fine-Gray competing risk model was adjusted for age, sex, hypertension, diabetes, congestive heart failure, chronic kidney disease, atrial fibrillation, stroke, anemia, low-density lipoprotein cholesterol, percutaneous coronary intervention, angiotensin-converting enzyme inhibitor or angiotensin receptor blocker, β-blocker, statins, and dual-antiplatelet therapy.


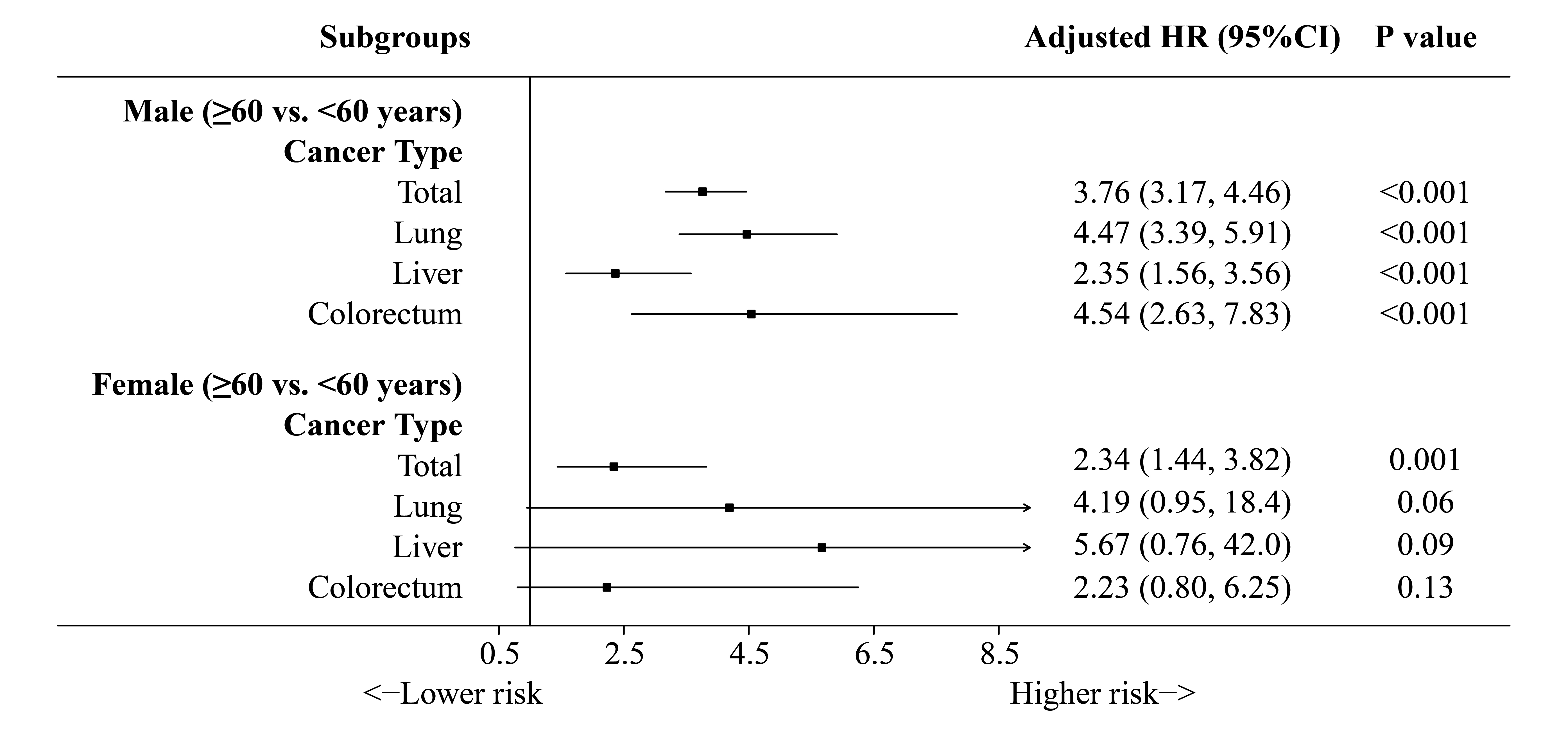
**Supplemental Figure 3** Hazard ratios (HR) and 95% confidence intervals (CIs) of cancer-specific mortality in sex and different age groups after missing value imputing.

**P* for interaction test: multiplicative interaction analysis between sex and different age groups (<60 and ≥60 years).

Fine-Gray competing risk model was adjusted for age, sex, hypertension, diabetes, congestive heart failure, chronic kidney disease, atrial fibrillation, stroke, anemia, low-density lipoprotein cholesterol, percutaneous coronary intervention, angiotensin-converting enzyme inhibitor or angiotensin receptor blocker, β-blocker, statins, and dual-antiplatelet therapy.


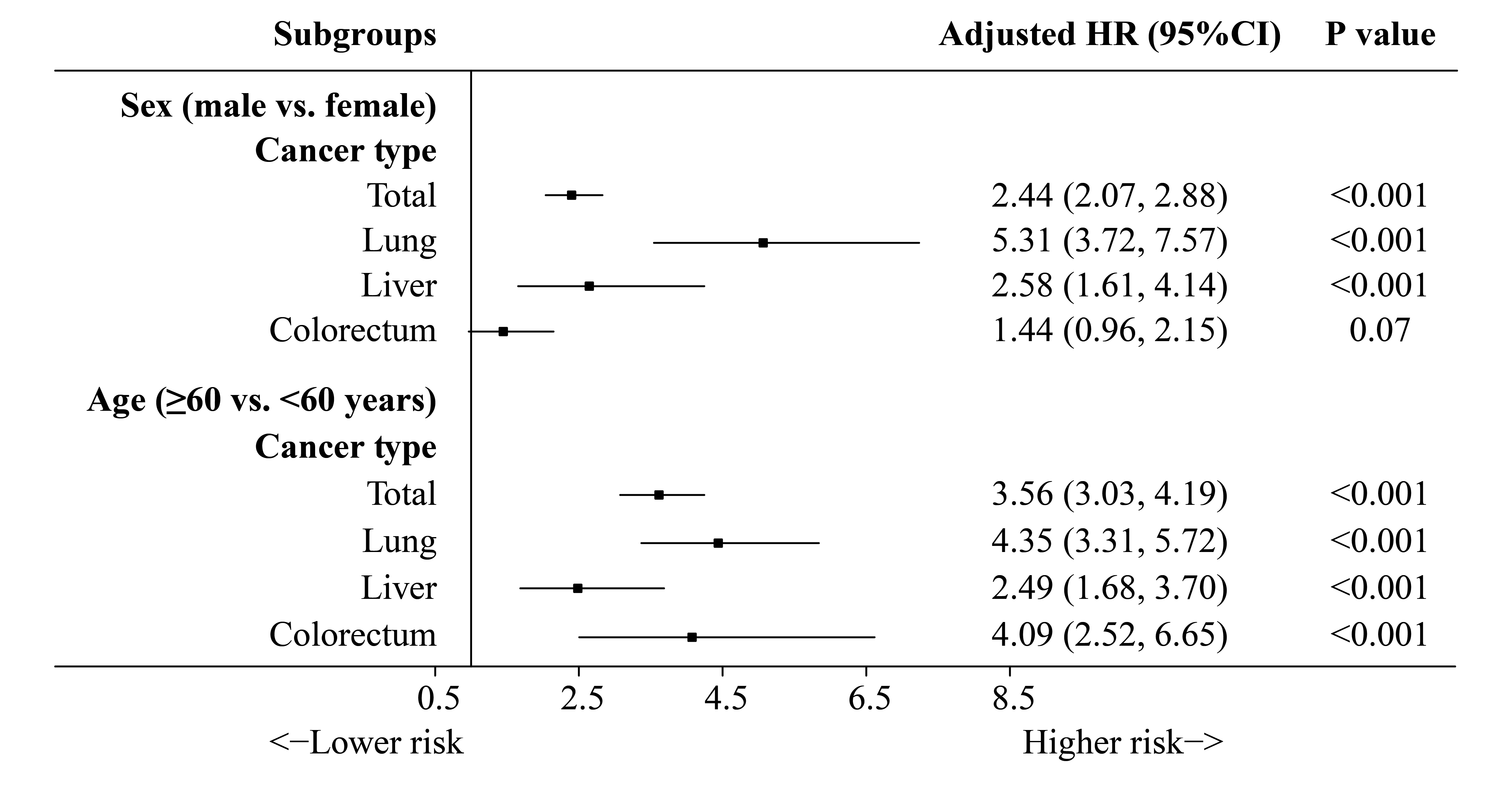
**Supplemental Figure 4** Hazard ratios (HR) and 95% confidence intervals (CIs) of cancer-specific mortality in sex different and further stratified by three age groups after missing value imputing.

**P* for interaction test: multiplicative interaction analysis between sex and different age groups (<60 and ≥60 years).

Total: *P* for interaction<0.001; Lung: *P* for interaction<0.001; Liver: *P* for interaction<0.001; *P* for interaction: Colorectum<0.001.

Fine-Gray competing risk model was adjusted for age, sex, hypertension, diabetes, congestive heart failure, chronic kidney disease, atrial fibrillation, stroke, anemia, low-density lipoprotein cholesterol, percutaneous coronary intervention, angiotensin-converting enzyme inhibitor or angiotensin receptor blocker, β-blocker, statins, and dual-antiplatelet therapy.
